# IL‐37b alleviates inflammation in the temporomandibular joint cartilage via IL‐1R8 pathway

**DOI:** 10.1111/cpr.12692

**Published:** 2019-09-27

**Authors:** Ping Luo, Chi Feng, Chao Jiang, Xiaochun Ren, Liming Gou, Ping Ji, Jie Xu

**Affiliations:** ^1^ College of Stomatology Chongqing Medical University Chongqing China; ^2^ Chongqing Key Laboratory for Oral Diseases and Biomedical Sciences Chongqing China; ^3^ Chongqing Municipal Key Laboratory of Oral Biomedical Engineering of Higher Education Chongqing China

**Keywords:** cartilage, IL‐1R8, inflammation, interleukin‐37, temporomandibular joint

## Abstract

**Objectives:**

Interleukin (IL)‐37 is a natural suppressor of innate inflammation. This study was conducted to explore the anti‐inflammatory effects of IL‐37 in temporomandibular joint (TMJ) inflammation.

**Materials and Methods:**

The expression of IL‐37 in the TMJ was measured using ELISA and IHC. Human TMJ chondrocytes were treated with IL‐37b and IL‐1β, and inflammation‐related factors were detected. siRNA‐IL‐1R8 was transfected into chondrocytes, and the affected pathways were detected. IL‐37b was used in disc‐perforation‐induced TMJ inflammation in SD rats. Micro‐CT, IHC, real‐time PCR and histological staining were used to quantify the therapeutic effect of IL‐37b.

**Results:**

IL‐37 was expressed in the synovium and the disc of patients with osteoarthritis (OA) and in the articular cartilage of condylar fracture patients. IL‐37 was highly expressed in synovial fluid of patients with synovitis than in those with OA and disc displacement and was closely related to visual analogue scale (VAS) score. In vitro, IL‐37b suppressed the expression of pro‐inflammatory factors. In addition, IL‐37b exerted anti‐inflammatory effects via IL‐1R8 by inhibiting the p38, ERK, JNK and NF‐κB activation, while silencing IL‐1R8 led to inflammation and upregulation of these signals. In disc‐perforation‐induced TMJ inflammation in SD rats, IL‐37b suppressed inflammation and inhibited osteoclast formation to protect against TMJ.

**Conclusions:**

IL‐37b may be a novel therapeutic agent for TMJ inflammation.

## 1 INTRODUCTION

The temporomandibular joint (TMJ) is a synovial joint that is frequently affected by osteoarthritis.^1^ Temporomandibular joint osteoarthritis (TMJOA), which is considered a prevalent chronic pain disease, leads to cartilage degeneration, subchondral bone remodelling and synovitis, thus influencing biopsychosocial conditions.^2^, ^3^ Inflammation is believed to be the chief cause of pain in patients with OA, resulting from the release of pro‐inflammatory cytokines.^4^ Increasing evidence indicates that inflammation plays a critical role in the pathogenesis of TMJOA.^5^, ^6^ Condition of TMJ disc displacement without restoration (DDWoR) is mainly characterized by mouth‐opening limitations.^7^ TMJ synovitis, the most common early‐detected inflammatory arthritis, always causes joint pain, especially during acute inflammation.^8^ IL‐1β is the major pro‐inflammatory cytokine that exhibits destructive effects that are characterized by increasing cartilage degradation and suppression of cartilage matrix synthesis.^9^, ^10^, ^11^ Excess production of matrix‐degrading proteases, including matrix metalloproteinases (MMPs) with disintegrin and metalloproteinase with thrombospondin motifs (ADAMTs), results from the disruption of metabolic homoeostasis and leads to cartilage degradation that occurs in inflammation.^9^ This leads to increased cleavage of aggrecan and production of collagen type I and type II molecules, which promotes cartilage destruction.^12^ There are several current treatments to relieve inflammation and prevent degradation of joint complex. However, the development of effective total joint treatments has been challenging.^3^, ^4^

Interleukin‐37 (IL‐37), a novel anti‐inflammatory cytokine, is a newly discovered member of the IL‐1 family and functions as a natural suppressor of inflammatory and immune responses.^13^ IL‐37 can be induced by inflammatory stimuli such as IL‐1β and LPS in PBMCs and synovial cells.^14^, ^15^ Five structurally related isoforms of human IL‐37 have been identified (IL‐37a‐e), with IL‐37b being the most complete and the most reported.^13^, ^16^ So far, a homologous gene for IL‐37 has not been identified in mouse. However, growing evidence confirms the effects of exogenous, recombinant IL‐37 on wild‐type (WT) mice subjected to calcific aortic valve disease,^17^ myocardial infarction,^18^ spinal cord injury^19^ and invasive pulmonary aspergillosis.^20^ Osteoclast recruitment might be central to diseases involving bone erosion and resorption.^21^ Saeed J reported that IL‐37 inhibits lipopolysaccharide‐induced osteoclast formation and bone resorption in mice.^22^ IL‐37 plays a crucial role in regulating inflammatory and autoimmune diseases,^13^, ^16^ including liver inflammation,^23^ arthritis^24^ and allergic airway inflammation.^25^

Despite fervent interest in IL‐37, which of the variants is expressed in the chondrocytes is unknown. The mechanisms underlying the effect of IL‐37 on the activation of signalling pathways remain unclear. In this study, we aimed to investigate the expressions of IL‐37 variants in chondrocytes and how IL‐37 might influence chondrocytes during inflammation. We focused on the effect of IL‐37 on disc‐perforation‐induced inflammation in SD rats in vivo. This study may provide a therapeutic target for the prevention and treatment of inflammation in osteoarthritis of the TMJ.

## 2 MATERIALS AND METHODS

### 2.1 Ethical statement for human sampling

Ethical approval was obtained from the Committee of Chongqing Medical University, and informed consent was signed by all participants before sample collection. The tissue and the synovial fluid samples were collected and processed in accordance with the Declaration of Helsinki.

### 2.2 Patients

Twenty TMJOA patients, 15 synovitis patients and 45 DDWoR patients with informed consent were recruited from the Stomatological Hospital of Chongqing Medical University. Each patient was diagnosed with cone beam computed tomography (CBCT) or MRI and clinical parameters. Clinical data from each patient were recorded. A visual analogue scale (VAS) was used to measure pain intensity (score 0‐10), with lower scores indicating lower levels of clinical symptoms or pain.^26^ Demographic and clinical information are listed in Table 1.

**Table 1:** Demographic and clinical characteristics of TMJOA, synovitis and DDWoR

Characteristics	Patients with OA (n = 20)	Synovititis (n = 15)	DDWoR (n = 45)
Age (mean ± SD)	47.650 ± 18.408	46.667 ± 22.908	43.731 ± 16.768
Male/Fmale	1/19	3/12	10/35
VAS score (mean ± SD)	5.250 ± 0.596	6.433 ± 0.903	3.000 ± 1.118

### 2.3 Sample collection and chondrocyte culture

The specimens of the synovium and disc were collected from patients with OA (n = 6), while condyles were collected from patients with condylar fracture (n = 5) for immunohistochemistry. Synovial fluid from 80 patients was collected during joint injection (20 OA, 15 synovitis and 45 DDWoR patients). The anterior part of the ear of each subject was sterilized. The upper cavity of the joint was injected with 1 mL physiological saline, and 1 mL diluent synovial fluid was extracted with no blood as described previously.^27^ The synovial fluid was centrifuged at 2000 r/min for 10 minutes. The supernatant was preserved at −80°C. These samples had undergone only one freeze‐thaw cycle before the study was carried out.

For cell culture, condyles were collected from six male patients between 21‐35 years old with the mean age of 28.3, with condylar fracture during surgery, and chondrocytes were immediately isolated from condylar cartilage. All cartilage specimens were washed with PBS and digested for 10 minutes in 0.25% trypsin (HyClone), followed by 1 mg/mL type I collagenase overnight. The chondrocytes were suspended in DMEM (HyClone) supplemented with 10% foetal bovine serum (HyClone) and 1% penicillin/streptomycin and cultured at 37°C in air containing 5% CO_2_. After seeding the cells in the culture plates, the chondrocytes were pre‐treated with human recombinant IL‐37b (0.1 ng/mL; SinoBiological) for 2 hours followed by stimulation with pro‐inflammatory factor IL‐1β (10 ng/mL). For gene expression, samples were harvested after 24 hours; for protein expression, samples were harvested at 48 hours; and for detecting phosphorylation pathway, cells were harvested at 30 minutes.

### 2.4 Transfection of siRNA‐IL‐1R8

Chondrocytes were transiently transfected with siRNA targeting IL‐1R8 (GenePharma) or negative‐control siRNA (scrambled; GenePharma) using GP‐siRNA‐mate‐plus transfection reagent (GenePharma) following the manufacturer's instructions. All experiments were performed 48 hours after transfection, and the most effective single siRNA was used for further experiments.

### 2.5 Enzyme‐linked immunosorbent assay (ELISA)

IL‐37 was measured by conventional ELISA (Shanghai Jianglai Biotech). The method was performed according to the manufacturer's protocol.

### 2.6 Western blotting

Total protein concentration was measured using an Enhanced BCA Protein Assay Kit (Beyotime). For Western blot analysis, 30 μg of cell lysates were loaded for each sample. The proteins were transferred to PVDF membranes and blotted with antibodies for MMP1 (1:1000; Bioss), MMP9 (1:800; Zen Bioscience), MMP13 (1:1000; Abcam) and p‐p38 (1:500; Zen Bioscience), p‐ERK (1:1000; CST) and p‐JNK (1:750; Zen Bioscience). GAPDH (1:2000; Bioss) was blotted as the loading control. Images were obtained and analysed by a computer program (image j). Blots are representative of three separate and reproducible experiments.

### 2.7 Quantitative real‐time polymerase chain reaction (qRT‐PCR)

The cells and tissues from TMJ were harvested and lysed in RNAiso plus (Takara). Total RNA was collected, and the total RNA was reverse‐transcribed using a PrimeScript^™^RT Reagent Kit with gDNA Eraser (Takara) to obtain cDNA. Next, Real‐time PCR was performed on a BIORAD real‐time PCR system (CFXConnect) with 40 cycles using Power TB green PCR Master Mix (Takara). GAPDH‐specific primers were used as an internal control. The expression of target genes was normalized to GAPDH and was reported using 2^−ΔΔct^ method. The primer sequences used for qRT‐PCR in this study are shown in Table 2.

**Table 2:** Human (h) and SD‐Rat (r) qRT‐PCR primers used in this study

Gene	Forward primer (5′‐3′)	Reverse primer (5′‐3′)
hIL‐37a	CCAAGCCTCCCCACCATGAA	TCCAGGACCAGTACTTTGTGATC
hIL‐37b	CTGCTTAGAAGGTCCAAAGGTGA	GCTATGAGATTCCCAGAGTCCAG
hIL‐37c	CCCAGTGCTGCTTAGAAGAGATCTTCT	GCTGAAGGGATGGATGACTTTGTCCT
hIL‐37d	CCAAGCCTCCCCACCATGAATT	ACTTCCTTTCTCCGCAGAGGCTG
hIL‐37e	ATGTCAGGCTGTGATAGGAGGGAA	GACCAGTACTTTGTGATCCTGGTCATG
hIL‐1β	AATCTGTACCTGTCCTGCGTGTT	TGGGTAATTTTTGGGATCTACACTCT
hIL‐6	AGCCCACCGGGAACGA	GGACCGAAGGCGCTTGT
hIL‐8	AGAAGTTTTTGAAGAGGGCTGAGA	AGTTTCACTGGCATCTTCACTGATT
hMMP1	ACTGCCAAATGGGCTTGAAG	TTCCCTTTGAAAAACCGGACTT
hMMP3	GAGGCATCCACACCCTAGGTT	TCAGAAATGGCTGCATCGATT
hMMP9	CCCTTGTGCTCTTCCCTGGA	TCTGCCACCCGAGTGTAACC
hMMP13	ATTAAGGAGCATGGCGACTTCT	CCCAGGAGGAAAAGCATGAG
hADAMTS4	GGTCAAGGTCCCATGTGCAAC	GAATGCGGCCATCTTGTCATC
hIL‐1R8	ATGTCAAGTGCCGTCTCAACG	GCTGCGGCTTTAGGATGAAGT
hGAPDH	CTTTGGTATCGTGGAAGGACTC	GTAGAGGCAGGGATGATGTTCT
rIL‐1β	GCTGTGGCAGCTACCTATGTCTTG	AGGTCGTCATCATCCCACGAG
rIL‐6	CCACTTCACAAGTCGGAGGCTTA	GTGCATCATCGCTGTTCATACAATC
rMMP9	AAATGTGGGTGTACACAGGC	TTCACCCGGTTGTGGAAACT
rMMP13	GTGACTCTTGCGGGAATCCT	CAGGCACTCCACATCTTGGT
rGAPDH	TGCACCACCAACTGCTTAG	GATGCAGGGATGATGTTC

### 2.8 Immunofluorescence

Cells were treated with IL‐1β (10 ng/mL) to induce the translocation of NF‐κB into the nucleus. Meanwhile, chondrocytes were pre‐treated with IL‐37b (0.1 ng/mL) or transfected with siRNA‐IL‐1R8. After 48 hours treatment, cells were fixed with 4% paraformaldehyde and blocked with goat serum for 1 hour. Next, cells were incubated with NF‐κB‐p65 antibody (1:400; CST) overnight, and with goat anti‐rabbit antibody (1:400; BIOSS) conjugated with Alexa Fluor 555 for 1 hour the next day. Cells were stained with DAPI. Images were acquired using Olympus microscope (Olympus).

### 2.9 In vivo TMJ inflammation model

All procedures and administrations for the animals were approved by Chongqing Medical University Ethics Committee and were performed according to the institutional guidelines. Sprague‐Dawley rats (SD rats) were ordered from Chongqing Medical University. Male Sprague‐Dawley rats (SD rats; 300‐350 g) of 12 weeks old were kept in the animal facility for at least 7 days before use and received food and water ad libitum. SD rats were (n = 36 total animals) randomly divided into three groups: a sham‐operated group (n = 12), model group (n = 12) and treatment group (n = 12). For TMJ disc perforation, an oblique incision was created along the zygomatic arch. Then, the TMJ superior joint space was exposed, and the disc was pulled out and perforated using a micro‐ball‐shaped burr with 1.5‐mm diameter at the posterolateral part of TMJ disc. Then, the treatment group was treated with a volume of 50 μL human recombinant IL‐37b (Sino Biological) at a dose of 20 µg/mL (twice per week). However, the disc of the sham group was not perforated. In all the groups, care was taken to prevent damage to the TMJ articular surfaces. Postoperative complications did not occur. SD rats were randomly euthanized at 1 week and 4 weeks (n = 18 for each time point) for further study. All sections of this report adhered to the ARRIVE Guidelines for reporting animal research.^28^

### 2.10 Histological staining

TMJ samples of all animals were harvested and fixed in 4% paraformaldehyde overnight after sacrifice. After decalcification in 10% EDTA (pH 7.2‐7.4), samples were embedded in paraffin and cut into 5 μm sections. Safranin O and fast green staining (Solarbio) was performed to determine proteoglycan changes, and the Osteoarthritis Research Society International (OARSI) grades were used in assessing bone destruction. The OARSI grade methods were described previously.^29^ Tartrate‐resistant acid phosphatase (TRAP) staining was performed according to a standard protocol (Njjcbio). Only those cells showing TRAP‐positive staining and comprising three or more nuclei were considered as osteoclasts. The numbers of TRAP‐positive osteoclasts in the posterior and middle condylar subchondral bone were determined.

### 2.11 Immunohistochemistry

Immunohistochemical (IHC) staining of IL‐37 (1:250; Abcam), type I collagen (1:250; Bioss), type II collagen (1:250; Bioss), MMP1 (1:250; Bioss), MMP9 (1:250; Zen Bioscience) and MMP13 (1:250; Abcam) was performed. Sections were blocked in 10% normal goat serum, and primary antibody was incubated overnight at 4°C. The next day, HRP‐conjugated secondary antibodies were used for detection after incubating the slides in 0.3% H_2_O_2_ in PBS for 15 minutes. PBS instead of the primary antibody was added to negative controls. Diaminobenzidine (DAB) was used as substrate for colour development. All slides were counterstained with haematoxylin. Images of histologically stained sections were obtained using an Olympus microscope (Olympus). An immunoreactivity scoring system was applied as described in previous studies.^30^ The mean optical densities of collagen I, collagen II, MMP1, MMP9 and MMP13 were obtained from the cartilage using image measurement software (image‐pro plus 6.0; Media Cybernetics).

### 2.12 Micro‐CT

The scans were performed using the following scanner settings: X‐ray source voltage, 70 kVp and current 114 μA, 8 W. CT scan (SCANCO Medical AG) settings were high resolution, voxel size of 15 μm, slice thickness of 0.01 mm and FOV/diameter of 30.7 mm, with an integration time of 250 ms Micro‐CT was used to assess the loss of subchondral bone. Bone volume fraction (BV/TV, 1), trabecular thickness (Tb.Th, mm), trabecular number (Tb.N, 1/mm) and trabecular separation (Tb.Sp, mm) of TMJ subchondral bone were analysed. The height of the joint, measuring from the highest point of absorption of the joint to the neck of the condyle, was used to evaluate the degree of articular cartilage degradation.

### 2.13 Statistical analysis

All quantitative data are expressed as the mean ± SD unless otherwise indicated. Statistical analyses were performed with spss 19.0 (IBM Corporation). Pearson's correlation coefficient was used to assess the correlation of IL‐37 in the synovial fluid with VAS score. The rest of the values are expressed as means ± SD. Data were examined for normality by skewness/kurtosis. Transforming a non‐normal distribution into a normal distribution was performed using Blom's formula.^31^ Significant differences in the results were determined by one‐way ANOVA with Tukey test. A *P* value < .05 was considered statistically significant. Ns indicates no significant difference*. P* < .05, *P* < .01, *P* < .001 and *P* < .0001 in figures were represented as *, ** *** and ****, respectively.

## 3 RESULTS

### 3.1 IL‐37 protein level is positively correlated with TMJ inflammation

**Figure 1:**
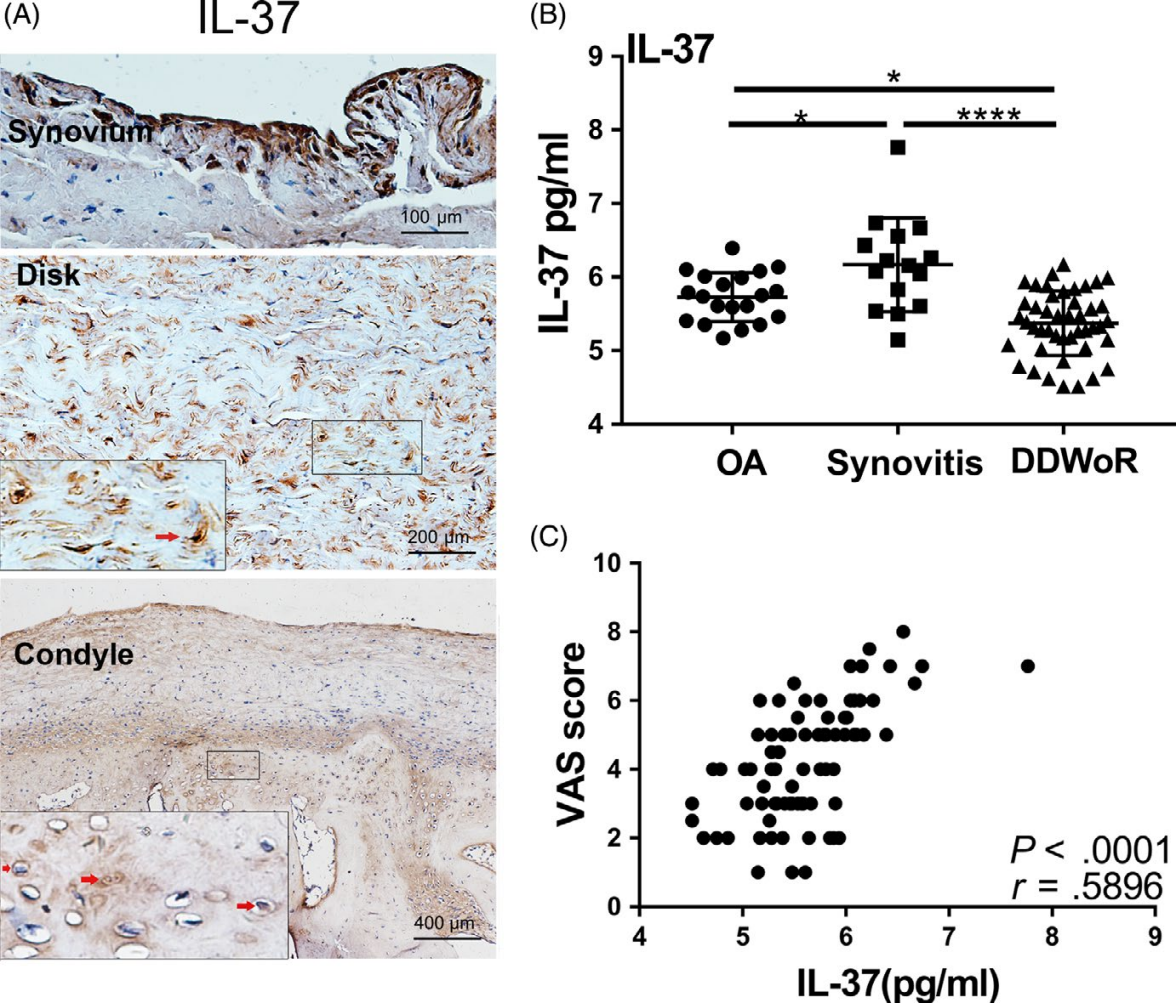
The expression of IL‐37 in TMJ. A, Immunohistochemical staining of IL‐37 in the synovium, disc and condylar cartilage. B, Levels of IL‐37 in synovial fluid of patients with TMJOA (n = 20), synovitis (n = 15) and DDWoR (n = 45) by ELISA. Data are expressed as the mean ± SD and analysed by one‐way ANOVA followed by Tukey's test. C, Correlations of synovial fluid IL‐37 level and VAS scores, n = 80. The correlations were evaluated with Pearson's correlation coefficient

To analyse the involvement of IL‐37 in human TMJ, the expression of IL‐37 in human inflammatory TMJ was first examined using immunohistochemistry (Figure 1A). IL‐37 was expressed in the inflammatory articular disc, the cartilage and the synovium of patients. IL‐37 was mainly localized in the nucleus and could be observed in the membranes, cytoplasm and extracellular matrix of TMJ cells. Patients with active TMJ synovitis showed higher IL‐37 level than OA and non‐acute inflammatory DDWoR patients (Figure 1B). As shown in Figure 1C, IL‐37 level in synovial fluid was positively correlated with VAS score (*r* = .5896, *P* < .0001).

### 3.2 IL‐37b suppresses IL‐1β‐induced expression of related genes in chondrocytes

**Figure 2:**
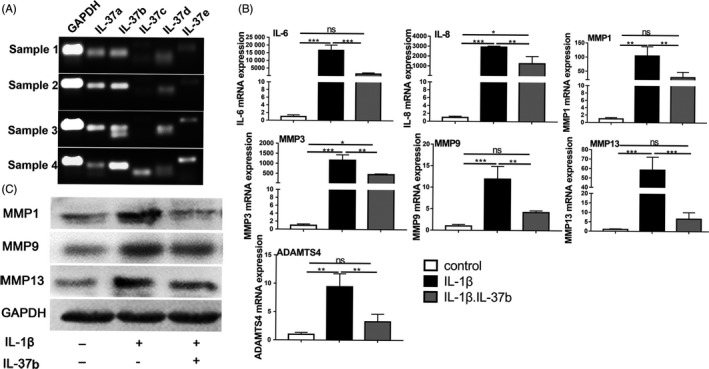
IL‐37b suppresses IL‐1β‐induced expression of inflammatory‐related genes in chondrocytes. A, RT‐PCR analysis of five splice variants of IL‐37 gene (IL37a–e) in the chondrocytes. B, The mRNA expression of IL‐6, IL‐8, MMP1, MMP3, MMP9, MMP13 and ADAMTS4 was determined by qRT‐PCR. C, MMP1, MMP9 and MMP13 protein expression levels in human chondrocytes pre‐treated with IL‐37b for 2 h followed by induction with IL‐1β for 48 h; one‐way ANOVA followed by Tukey's test. *P* values < .05 were considered significant, n = 3. All data are expressed as the mean ± SD

To examine which variants of IL‐37 are expressed in the chondrocytes, RT‐PCR was performed to measure IL‐37a‐e mRNA expression levels in the chondrocytes. Condylar chondrocytes produced higher levels of IL‐37a and b mRNAs but tiny amount of IL‐37c, d and ‐e mRNAs (Figure 2A). Next, to determine the anti‐inflammatory effect of IL‐37b, chondrocytes were pre‐treated with recombinant IL‐37b for 2 hours, followed by IL‐1β stimulation. IL‐37b significantly alleviated IL‐1β‐induced inflammatory gene expression of IL‐6, IL‐8, MMP1, MMP3, MMP9, MMP13 and ADAMTS4 (Figure 2B). Similar results were obtained for protein analysis of MMP1, MMP9 and MMP13 (Figure 2C).

### 3.3 Attenuation of IL‐37 activity in silencing IL‐1R8

**Figure 3:**
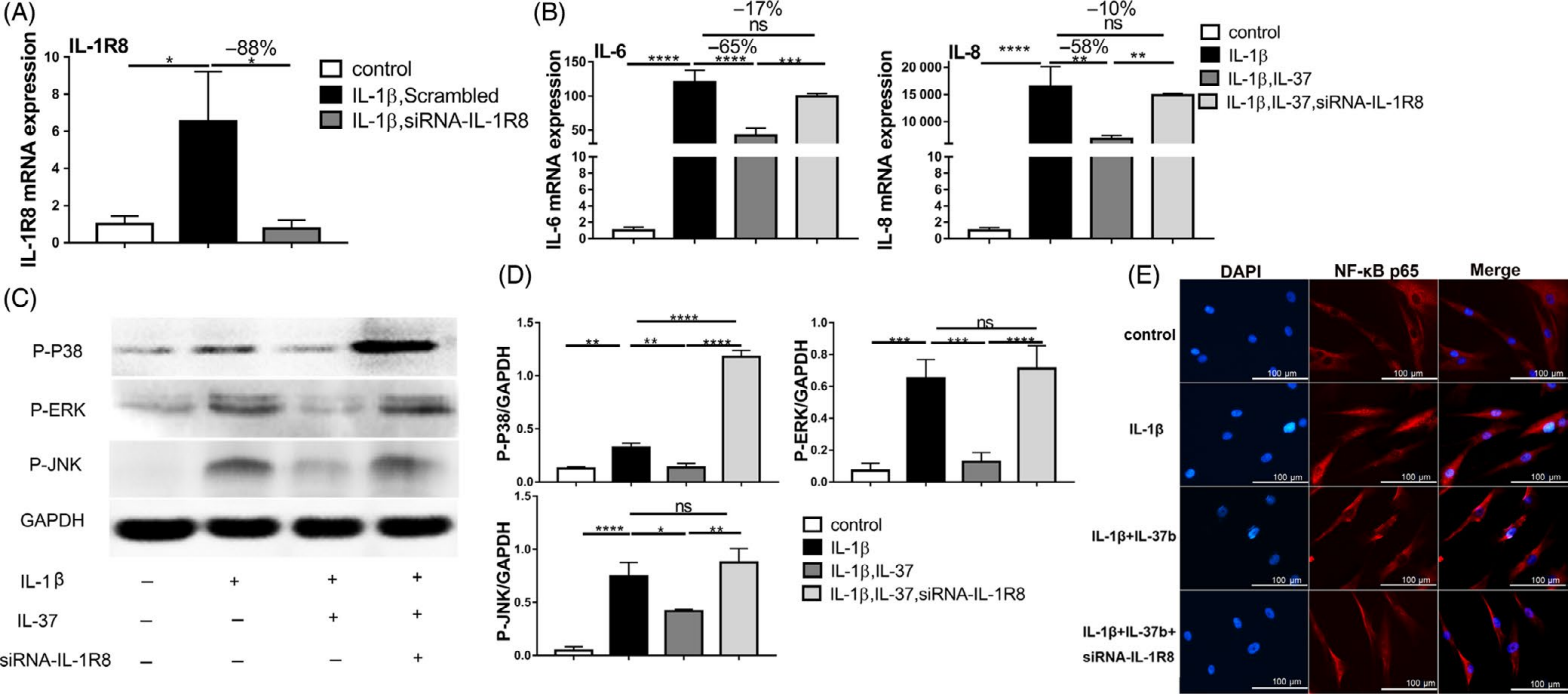
IL‐37b reduces the activation levels of MAPKs and NF‐κB p65 in an IL‐R8 dependent manner. A, Chondrocytes were transfected with small interfering RNA (siRNA) for IL‐1R8 or negative control siRNA (scrambled), and IL‐1R8 mRNA levels were analysed by qRT‐PCR. B, After transfection with IL‐1R8 siRNA or negative control siRNA for 48 h, chondrocytes were incubated with or without IL‐37b (0.1 ng/mL) for 2 h followed by stimulation with IL‐1β, and gene expression levels of IL‐6 and IL‐8 were then assessed by qRT‐PCR at 24 h. C, The p‐p38, p‐ERK and p‐JNK were detected by Western blot. D, Semi‐quantitative analysis of Western blot. E. NF‐κB p65 translocation was determined by immunofluorescence. *P* values < .05 were considered significant, n = 3. One‐way ANOVA followed by Tukey test. All data are expressed as the mean ± SD

To explore whether IL‐37b recruits IL‐1R8 for exerting anti‐inflammatory effects, IL‐1R8‐specific siRNA was constructed to knock‐down IL‐1R8 gene in vitro. qRT‐PCR indicated that chondrocytes transfected with siRNAs had decreased expression of IL‐1R8 compared to the controls (scrambled; Figure 3A). After pre‐treatment with IL‐37b for 2 hours and stimulation with IL‐1β, the abundance of pro‐inflammatory IL‐6 (−65%) and IL‐8 (−58%) was diminished. In the absence of IL‐1R8, the anti‐inflammatory effect of IL‐37b was obviously weaker, the decrease in IL‐6 level was only 17% (in contrast to the 65% noted above) and that in IL‐8 was 10% (in contrast to the 58% noted above; Figure 3B).

### 3.4 Recombinant IL‐37b inhibits p38, JNK, ERK and NF‐κB p65 activation in response to IL‐1β association with IL1R8

To further explore the possible effect of IL‐37b‐IL‐1R8 complex on MAPK pathway, the lysates in the phospho‐kinase array were assessed by Western blotting. As shown in Figure 3C and Figure 3D, exposure to IL‐1β increased p‐ERK, p‐p38 and p‐JNK levels compared to those in non‐stimulated cells. However, in IL‐37‐pre‐treated cells, p‐ERK, p‐p38 and p‐JNK levels were significantly attenuated. After silencing IL‐1R8 in the chondrocytes, levels of these signals were significantly reactivated. Next, the activation of NF‐κB p65 was measured via immunofluorescence. As shown in Figure 3E, cells were treated with IL‐1β to induce NF‐κB p65 translocation from the cytoplasm to the nucleus. Pre‐treatment with IL‐37b inhibited the translocation of NF‐κB into the nucleus; however, silencing IL‐1R8 resulted in failure to inhibit the translocation of NF‐κB into the nucleus.

### 3.5 IL‐37b alleviates condyle degradation in disc‐perforated TMJ

**Figure 4:**
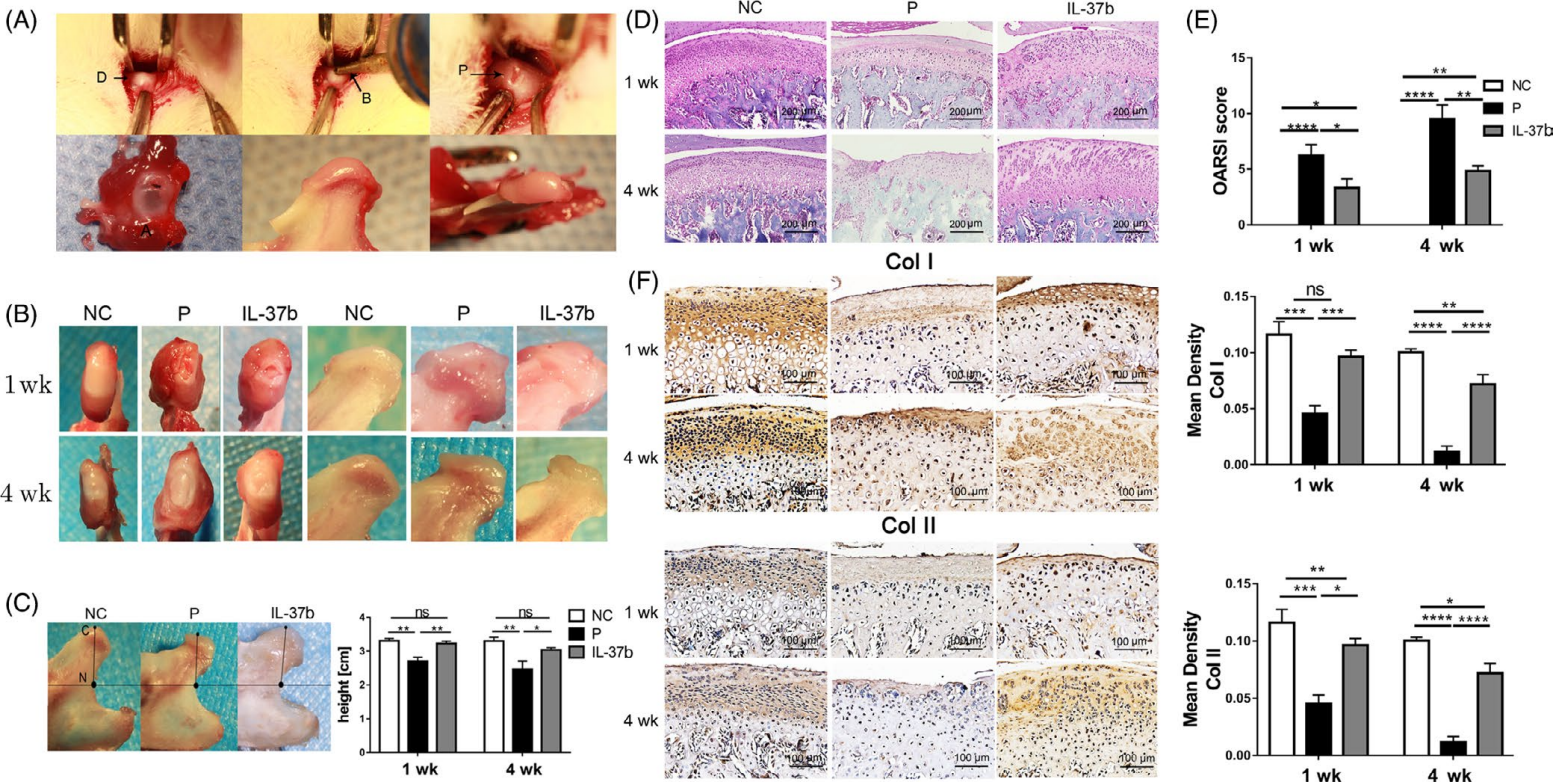
IL‐37b alleviates condyle degeneration in disc‐perforation‐induced inflammation in SD rats. A, The surgical procedure of disc perforation. D: disc, B: ball‐shaped burr, P: perforation, A: anterior disc. B, Representative morphological features of the condyle in control, model and treatment group. C, Condylar height changes in the three groups. C: condylar surface; N: condylar neck. D, Safranin O and fast green staining analyses of glycosaminoglycan in the TMJ. E, Cartilage layers OARSI grades. F, Immunohistochemical analyses of Col I and Col II in the condylar cartilage. The *P* values < .05 were considered significant; one‐way ANOVA followed by Tukey's test, n = 6 per group. All data are expressed as the mean ± SD. NC: negative control group. P: disc perforation model group. IL‐37b: treatment group

**Figure 5:**
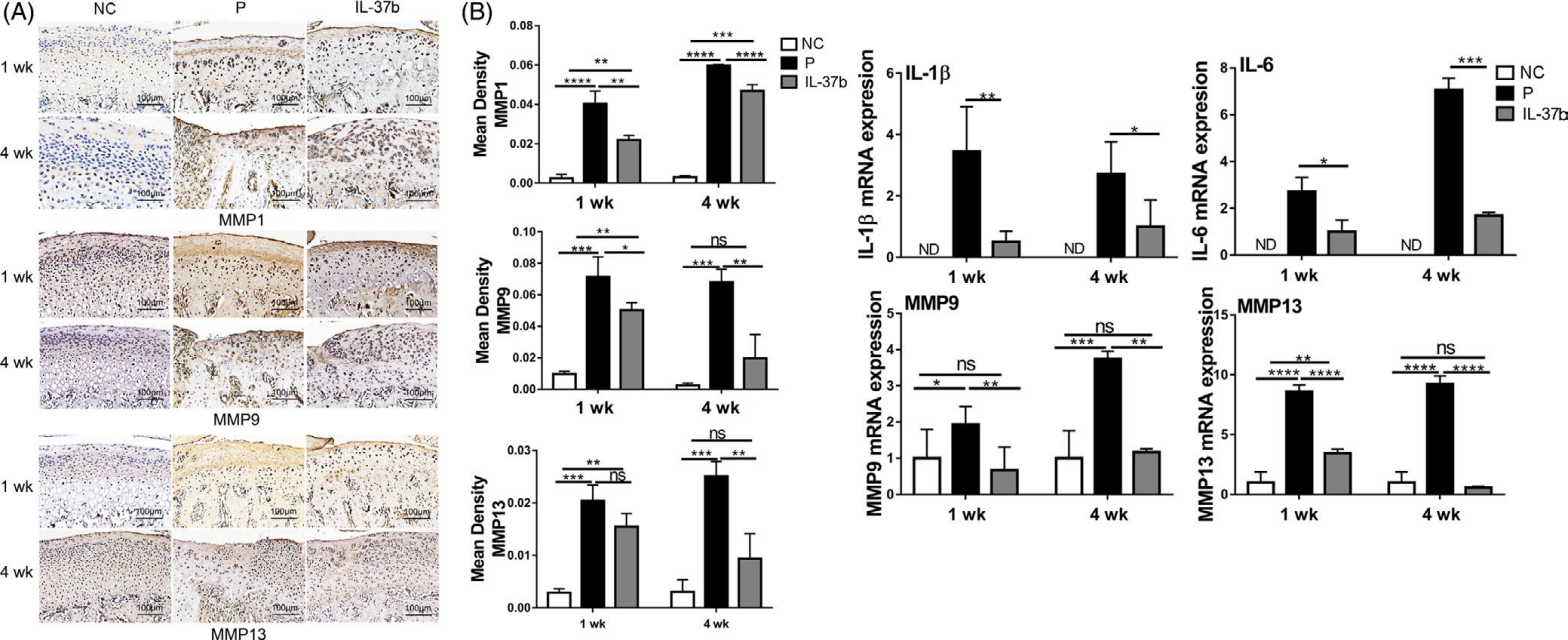
IL‐37b protects condyle degradation by suppressing pro‐inflammatory cytokine expression. A, Immunohistochemical analyses of MMP1, MMP9 and MMP13 in the condylar cartilage (n = 6). B, The mRNA expression of IL‐1β, IL‐6, MMP9 and MMP13 in condylar cartilage was analysed by RT‐qPCR *P* values < .05 were considered significant; one‐way ANOVA followed by Tukey test, n = 6 per group. All data are expressed as the mean ± SD

To further assess the contribution of IL‐37b to anti‐inflammatory functions in vivo, a disc‐perforation‐induced inflammation model was established in SD rats (4A). Macroscopic analysis showed that the size of disc perforation in model group was larger, while the size of disc perforation in the IL‐37b‐treated group was smaller than that in the model group. In addition, there was a significant difference in macroscopic tissue reactions, amelioration of inflammation, swelling and hyperplasia between IL‐37b‐treated group and model group at all tested time points. Moreover, cartilage became significantly thinner and flatter after surgery, while IL‐37b‐treated group showed less change in the cartilage than the control group (Figure 4B). As shown in Figure 4C, we observed that the height of the condyle in the treatment group increased compared to the model group. The articular cartilage improvement in IL‐37b group was further confirmed in the histological assessment. Histopathological evidence showed that, compared to model group, the treatment group exhibited less cartilage damage and condyle erosion. In the model group, proteoglycan loss was remarkably observed with Safranin O, showing greatly reduced cartilage staining. In contrast, IL‐37b‐treated rats showed limited proteoglycan loss (Figure 4D). The inflammation‐induced cartilage destruction was evaluated using the modified OARSI grade system. The OARSI grade of IL‐37b treatment group was lower than that of the model group, and the normal group showed no cartilage destruction (OARSI grade 0; Figure 4E). To detect cartilage matrix loss, type I collagen (COL I) and type II collagen (COL II) were stained by immunohistochemistry, which showed that the treatment group had more expression of Col I and Col II in the cartilage layer compared to the model group (Figure 4F). In addition, immunohistochemistry showed significantly upregulated MMP1, MMP9 and MMP13 in the cartilage layer of the model group, while in IL‐37b treated group, there was significant downregulation of these inflammatory proteins (Figure 5A). Similarly, IL‐37b significantly reduced mRNA levels of pro‐inflammatory molecules IL‐1β, IL‐6, MMP9 and MMP13 (Figure 5B).

### 3.6 IL‐37b reduces subchondral bone loss in disc‐perforated TMJ

**Figure 6:**
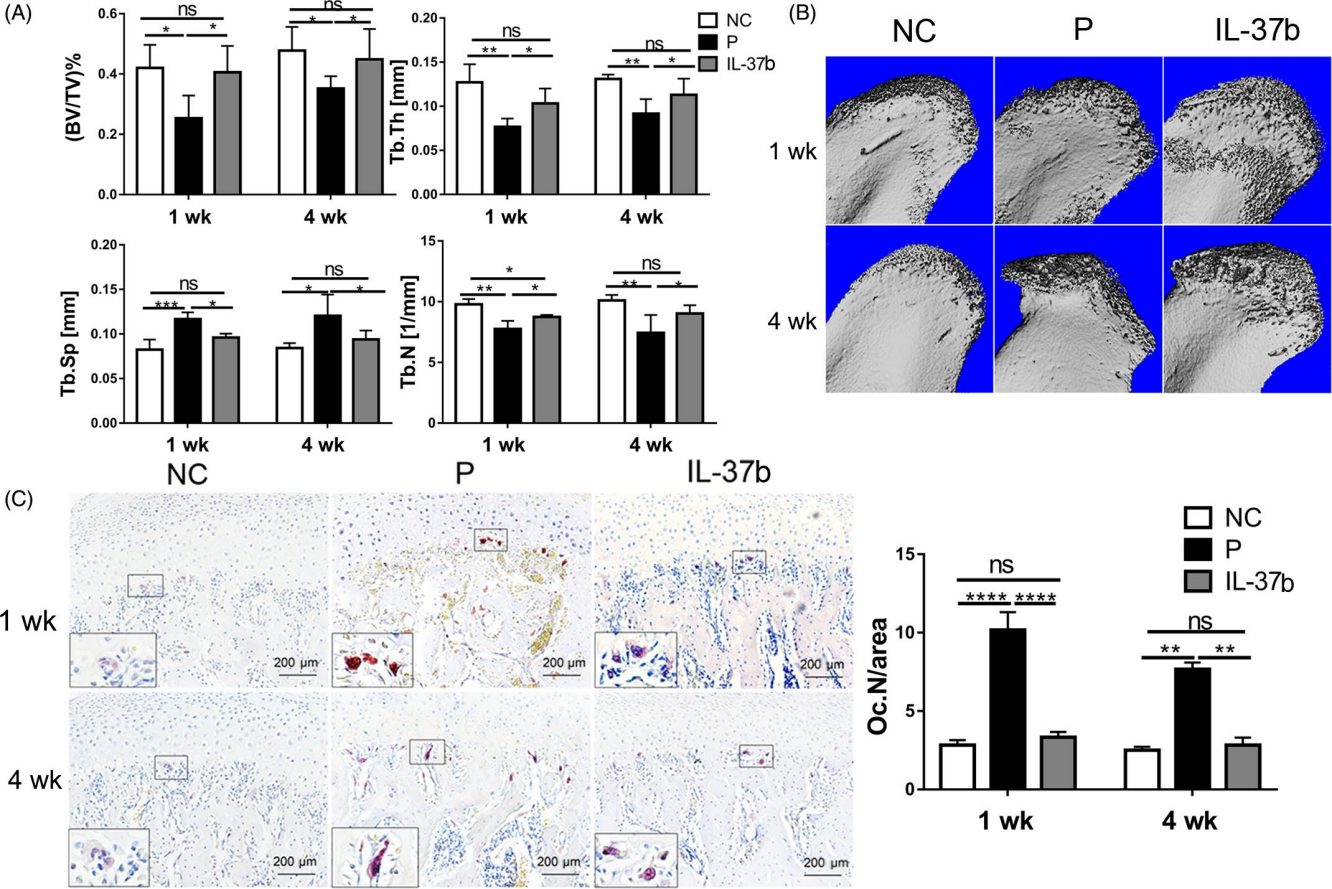
IL‐37b reduces subchondral bone loss in disc‐perforation TMJ. A, Quantitative analysis of the structural parameters of subchondral bone by micro‐CT. B, Three‐dimensional image of the condyle. C, TRAP staining of the TMJ subchondral bone and quantitative analysis. OC.N/area: the number of TRAP‐positive cells (per 400 × magnification). *P* values < .05 were considered significant; One‐way ANOVA followed by Tukey's test. All data are expressed as the mean ± SD, n = 6 per group

Micro‐CT was used to evaluate the changes in the microarchitecture of TMJ subchondral bone. Figure 6A showed that the model group had a lower bone volume fraction (BV/TV), trabecular thickness (Tb.Th) and trabecular number (Tb.N) than the control group. However, IL‐37b‐treated group showed a statistically significant increase in BV/TV, Tb.Th and Tb.N in subchondral trabecular bone compared to the model group. However, trabecular separation (Tb.Sp) in the model group was higher than that in the control group and significantly lower in IL‐37‐treated group than in the model group. The three‐dimensional images showed subchondral bone loss, and the treatment group showed significantly less subchondral bone loss than the model group, while the control TMJ maintained subchondral bone integrity (Figure 6B). In addition, the number of tartrate‐resistant acid phosphatase (TRAP)‐positive cells, as a biomarker for bone resorption in the subchondral bone was significantly decreased in IL‐37b‐treated rats (Figure 6C).

## 4 DISCUSSION

Osteoarthritis is the most common joint disease in the world, but there is no standard treatment to prevent OA progression. However, increasing evidence shows that inflammation plays a critical role in OA pathology, suggesting that targeting inflammation in OA could be a promising strategy.^6^, ^7^ IL‐37 has emerged as a fundamental inhibitor of the innate immune response, attenuating inflammation in models of periodontal inflammation,^32^ gout^33^ and colitis.^34^ However, no study has investigated the effect of IL‐37 on TMJ inflammation. Based on these findings, we investigated whether and how IL‐37 downregulates TMJ inflammation.

First, to analyse the involvement of IL‐37 in human TMJ inflammation, our data revealed that IL‐37 was expressed in the inflamed disc and synovium of patients with TMJOA and condyle cartilage in condylar fracture. In the synovial fluid, IL‐37 was highly expressed in active inflammation in patients with synovitis than in those with OA and DDWoR. Unfortunately, for ethical reasons, healthy individuals were not included. In addition, significant positive correlations were observed between synovial fluid IL‐37 level and clinical VAS score.

Next, we studied the anti‐inflammatory properties of the recombinant human IL‐37b in TMJ chondrocytes in vitro. IL‐37 possesses five different isoforms (named IL‐37a–e).^13^, ^16^ However, which isoform is expressed in the TMJ chondrocytes remains unknown. Interestingly, our study suggested that IL‐37a and IL‐37b were highly expressed in the chondrocytes. It has been reported that IL‐37b being the most functional complete of these isoforms, and the anti‐inflammatory effect was the most widely reported.^13^ In addition, IL‐37b was the most abundant in the chondrocytes, so we focused on this isoform. Recombinant IL‐37b inhibited the expression of inflammatory cytokines, such as IL‐6, IL‐8, MMPs and ADAMTS4, in IL‐1β‐induced inflammation in vitro, consistent with previously published study.^14^, ^35^, ^36^

To explore the specific mechanism of action of IL‐37b in inflammation, it was important to know the mechanism through which receptor complex IL‐37b works. IL‐37b can bind to IL‐1R8 to involve in allergic airway inflammation,^25^ asthma^35^, ^36^ and atherosclerosis.^37^ Therefore, we hypothesized that IL‐1R8 is required for IL‐37b to downregulate inflammation in TMJ. Here, IL‐1R8 was knocked‐down and assessed by qRT‐PCR. This clearly demonstrated that the absence of IL‐1R8 almost completely abolished IL‐37b protection against IL‐1β‐induced inflammation. IL‐1β is the major pro‐inflammatory cytokine implicated in TMJOA, and it activates the signalling cascade involving NF‐κB and the cascades involving the terminal MAPKs JNK, p38 and ERK.^6^ It has been reported that IL‐37 suppresses LPS‐induced inflammation in macrophages by downregulating JNK, p38 and ERK signalling pathways.^38^, ^39^ In addition, some reports showed that IL‐37 inhibited inflammation by inhibiting NF‐κB activation.^40^ Our data revealed that in the chondrocytes, IL‐37b downregulated ERK, JNK and p38 MAPK signals and inhibited NF‐κB translocation into the nucleus after IL‐1β stimulation. In contrast, silencing IL‐1R8 led to inflammation upregulation by enhancing these signals. These results indicate that IL‐37b inhibits p38, JNK, ERK and NF‐κB P65 activation in response to IL‐1β association with IL1R8 to inhibit inflammation.^38^, ^39^ Summarily, these results suggest that local expression of IL‐37b contributes directly to the suppression of joint inflammation in TMJ and that IL‐37 requires IL‐1R8 to reduce inflammation by downregulating MAPK signals. These findings show that IL‐37b can be considered for treatment strategies in patients with TMJ inflammation.

The in vivo model of rat articular disc perforation showed that human recombinant IL‐37b protein reduced the expression of inflammatory factors and dampened the pathological process of inflammation in TMJ. Discectomy results showed OA‐like changes in rat TMJ,^11^, ^41^ and the same effect has been verified in rabbit joint disc perforation study.^42^ Sustained inflammation induces structural abnormalities in the TMJ.^11^ In our study, flattening of condyles, erosion and sclerosis were observed in the model group. The height of the condyle was more reduced in the model group than the treatment group, indicating that IL‐37b protected the joint and reduced bone destruction. Collagen constitutes a major proportion of the extracellular matrix (ECM) in the cartilage, and the degradation of the ECM is done mainly by MMPs.^43^ Therefore, to visualize proteoglycans and collagen protein changes in cartilage, the collagen content of condyles was measured by IHC and Safranin O and fast green staining, and the results showed that the collagen content in IL‐37b treatment group was higher than that in the model group. The inflammatory factors IL‐1β, IL‐6 and MMPs were significantly reduced in IL‐37b compared to the model group.^44^ Our in vivo results further indicated that IL‐37b had potent anti‐inflammatory effects and protected against TMJ inflammation by reducing collagen degradation and matrix metalloenzymes (MMPs). The production of osteoclasts is one of the important pathological features of inflammatory bone destruction. Severe inflammatory bone destruction is closely related to bone metabolism disorders.^45^ In our study, IL‐37b suppressed osteoclast formation. Tartrate‐resistant acid phosphatase (TRAP) staining showed that the positive cells in the treatment group were significantly fewer than that in the model group. This finding is consistent with that of micro‐CT, which indicated that L‐37b protected against subchondral bone loss in vivo. These results suggest that IL‐37b inhibits excessive inflammation in the TMJ and reduces joint destruction.^46^, ^47^

In conclusion, we demonstrated the therapeutic potential of the novel anti‐inflammatory cytokine IL‐37b in the treatment of inflammation of the TMJ. IL‐1R8 is required for IL‐37b to exert anti‐inflammatory effects in the TMJ. IL‐1R8 silencing impaired the activity of IL‐37b and upregulated inflammatory factors. Disc‐perforation‐induced inflammation revealed the anti‐inflammatory and condyle‐protective ability of IL‐37. These results suggest that targeting IL‐37 pathway may provide a new therapeutic strategy for treating human TMJ inflammation.

## CONFLICT OF INTEREST

The authors declare no conflicts of interest.

## Data Availability

The datasets generated and/or analysed during the current study are available from the corresponding author on reasonable request.
